# An Iterative Solver in the Presence and Absence of Multiplicity for Nonlinear Equations

**DOI:** 10.1155/2013/837243

**Published:** 2013-12-22

**Authors:** Fazlollah Soleymani, Stanford Shateyi, Gülcan Özkum

**Affiliations:** ^1^Young Researchers and Elite Club, Zahedan Branch, Islamic Azad University, Zahedan, Iran; ^2^Department of Mathematics, University of Venda, Private Bag X5050, Thohoyandou 0950, South Africa; ^3^Department of Mathematics, Science and Letter Faculty, Kocaeli University, Umuttepe Campus, Kocaeli, Turkey

## Abstract

We develop a high-order fixed point type method to approximate a multiple root. By using three functional evaluations per full cycle, a new class of fourth-order methods for this purpose is suggested and established. The methods from the class require the knowledge of the multiplicity. We also present a method in the absence of multiplicity for nonlinear equations. In order to attest the efficiency of the obtained methods, we employ numerical comparisons alongside obtaining basins of attraction to compare them in the complex plane according to their convergence speed and chaotic behavior.

## 1. Introduction

There are several methods for computing a zero x* with multiplicity *m* of a nonlinear equation *f*(*x*) = 0; see, for example, [1–4]. Newton's iterative method is only of first order unless it is modified to obtain the second order of convergence. This modification requires knowledge of the multiplicity. Traub in [5] has suggested the use of any method for *f* ^(*m*)^(*x*) or *h*(*x*): = *f*(*x*)/*f*′(*x*). Any such method will require higher derivatives than the corresponding one for simple zeros. In general, constructing iterative methods for finding multiple zeros is not an easy task and careful attention is required in their developments.

This work tries to deal with the solution of nonlinear equations when the multiplicity of the roots is available or can be approximated and also when *m* is very high or not available. It then will be clear that for the choice *m* = 1, the corresponding method can easily be applied for simple zeros.

In this paper, we consider first the following class of methods for simple zeros (with *c*_*k*_ = *f* ^(*k*)^(x*)/*k*!*f*′(x*)), (*k* = 2,3, 4):

(1) yn=xn−23f(xn)f′(xn),xn+1=yn−2f(xn)+(yn−xn)(f′(yn)+f′(xn))2f′(yn)×{G(tn)},

where *t*_*n*_ = *f*′(*y*_*n*_)/*f*′(*x*_*n*_), *G*(1) = 1, *G*′(1) = −1/4, and *G*′′(1) = 7/4 with the following error equation:

(2) en+1=1243×(−243c2c3+27c4+c23(399+32G(3)(1)))en4+O(en5).

We then extend (1) for multiple zeros without lowering the convergence order or applying much more computational evaluations of the function or derivatives. Thus, the proposed scheme will be of order four for multiple zeros with two evaluations of the first-order derivative and one evaluation of the function, which clearly implies the consistency of the general solver with the optimality conjecture of Kung-Traub [6]. Furthermore, we will discuss some qualitative issues using the attraction basins as a criterion for comparison. To this end, we refer the readers to see [7–9] on obtaining a background on this matter regarding a rational map on the Riemann sphere, Julia set, Newton's fractals, the definitions of dynamic and chaotic behaviors, and so forth.

The remaining sections of the paper are organized as follows.  Section 2 will shortly report some of the existing methods for multiple roots using three functional evaluations per full step. It will be followed by Section 3 wherein the main contribution of the paper lies, as a general fourth-order solver in the presence and the absence of multiplicity for nonlinear equations.  Section 4 will be devoted to the comparison of different methods based on basins of attraction to observe the chaotic behaviors of the corresponding fractals produced by each iterative method in the complex plane. Numerical reports will be furnished in Section 5 and the conclusion of the study has been presented in the last section.

## 2. A Brief Review

Here, we mention some of the famous schemes for multiple zeros using three functional evaluations per full cycle shortly. The old and well-known third-order method of Dong in [10] can be given as

(3) $$\begin{array}{c} y_{n}=x_{n}-\sqrt{m}\frac{f\left(x_{n}\right)}{f^{\prime }\left(x_{n}\right)},\\ x_{n+1}=y_{n}-m{\left(1-\frac{1}{\sqrt{m}}\right)}^{1-m}\frac{f\left(y_{n}\right)}{f^{\prime }\left(x_{n}\right)}. \end{array}$$

Dong has also proposed the following third-order method in [10]:

(4) yn=xn−f(xn)f′(xn),xn+1=yn −f(xn)(m/(m−1))m+1f′(yn)+(m−m2−1)/(m−1)2f′(xn).

In 2010, J. R. Sharma and R. Sharma in [11] presented the following quartically convergent method:

(5) yn=xn−2mm+2f(xn)f′(xn),xn+1=xn−m8×((m3−4m+8)−(m+2)2(mm+2)mf′(xn)f′(yn)×(2(m−1)−(m+2)(mm+2)mf′(xn)f′(yn)))×f(xn)f′(xn).

Recently in 2011, Zhou et al. in [12] presented the following optimal fourth-order scheme:

(6) yn=xn−2mm+2f(xn)f′(xn),xn+1=xn−m8((m3+6m2+8m+8)+m3(m+2m)2m(f′(yn)f′(xn))2−2m2(m+3)(m+2m)mf′(yn)f′(xn))f(xn)f′(xn).

All the reported methods above require the multiplicity of the zeros and three functional evaluations per computing step to proceed. Clearly, only methods (5) and (6) are optimal in the sense of Kung-Traub for constructing multipoint methods without memory for solving nonlinear equations. Some other new developments for multiple zeros can be found in [13, 14].

## 3. A New General Solver for Multiple Zeros

Let us consider the following class for multiple zeros as a generalized form of (1) and by using weight function approach:

(7) yn=xn−2mm+2f(xn)f′(xn),xn+1=yn−2f(xn)+(yn−xn)(f′(yn)+f′(xn))2f′(yn)×{H(τn)}, τn=f′(yn)f′(xn).

We now show that this class is of fourth order for multiple roots under certain conditions on the weight function.

**Theorem 1**

Let x* ∈ *D* be a multiple zero of a sufficiently differentiable function *f* : *D* ⊂ ℝ → ℝ for an open interval *D* with the multiplicity *m*, which includes *x*_0_ as an initial approximation of x*. Then, the class of methods without memory (7) is of optimal local order four, when

(8) H(p(m−1)/m)=−m(2+m)p−2+2p+mp,H′(p(m−1)/m)=14m2(−2m(4+m)+(2+m)3p)(−2+(2+m)p)2,H′′(p(m−1)/m)=−14m3(4m(2+m)−4m(8+m(5+m))p+(2+m)4p2)p(−2+(2+m)p)3,

and |*H* ^(3)^(*p* ^(*m* − 1)/*m*^)| < *∞*, where *p* = (*m*/(*m*+2))^*m*^.

**Proof**

To find the asymptotic error constant of (7) where *C*_*j*_ = (*m*)!/(*m* + *j*)! × *f* ^(*m*+*j*)^(x*)/*f* ^(*m*)^(x*), *j* ≥ 1, we expand any terms of (7) around the multiple zero x* in the *n*th iterate whence *e*_*n*_ = *x*_*n*_ − x*. Clearly, for such symbolic computations, a Computer Algebra System such as [15] needs to be used.

A Taylor expansion around x* yields *f*(*x*_*n*_) = (*f* ^(*m*)^(x*)/*m*!)*e*_*n*_^*m*^(1 + ∑_*j*=1_^*∞*^ *C*_*j*_ *e*_*n*_^*j*^) and *f*′(*x*_*n*_) = (*f* ^(*m*)^(*x*_n_*)/(*m* − 1)!)*e*_*n*_^*m*−1^(1 + ∑_*j*=1_^*∞*^((*m* + *j*)/*m*)*C*_*j*_ *e*_*n*_^*j*^), so that, using algebraic software again,

(9) yn=xn−2mm+2f(xn)f′(xn)=x∗+mm+2en+2C1m(m+2)en2 −((m+1)C12−2mC2)m2(m+2)en3 +2((−3m2−4m)C1C2+(m2+2m+1)C13+3m2C3)m3(m+2) ×en4+O(en5).

Now we easily have *f*′(*y*_*n*_) = *e*_*n*_^*m*^((*f*′(*α*)(*m*/(2 + *m*))^*m*^(2 + *m*))/(*m*!*e*_*n*_)+(*C*_1_ *f*′(*α*)(*m*/(2 + *m*))^*m*^(−4 + 2*m* + 3*m* ^2^ + *m* ^3^))/(*m* ^2^ *m*!)+(*f*′(*α*)(*m*/(2 + *m*))^*m*^(−4*C*_1_^2^(−2 + *m*) + *C*_2_ *m* ^2^(−8 + 4*m* + 4*m* ^2^ + *m* ^3^))*e*_*n*_)/(*m* ^4^ *m*!) + *O*(*e*_*n*_^2^)). Furthermore, we have by Taylor expansion

(10) τn=f′(yn)f′(xn)=p(m−1)/m−4pm3C1en+(4(m2+2)pm5C12−8pm3C2)en2+(−8(m4−m3+5m2+m+6)p3m7C13+8(m2+4)pm5C1C2−8(m2+6m+6)pm3(m+2)C3)en3+O(en4).

Thus by using (10), we obtain

(11) H(τn)=H(p(m−1)/m)+H′(p(m−1)/m)(τn−p(m−1)/m) +H′′(p(m−1)/m)2(τn−p(m−1)/m)2 +H(3)(p(m−1)/m)6(τn−p(m−1)/m)3 +O(en4)=−m(2+m)p−2+2p+mp −C1(−2m2p+6p2m2+p2m3+12p2m−8mp+8p2)m(−2+2p+mp)2 ×en+⋯+O(en5).

And also,

(12) 2f(xn)+(yn−xn)(f′(yn)+f′(xn))2f′(yn)=(−−2+2p+mpp(2+m)2)en +(C1(−2m2+4m2p+m3p+4mp+8−4m)p(2+m)3m2)en2 +1m3(m+2)4 ×(−C12m5+2m5C2+12C2m4−4C2m4p−7C12m4+2C12m4p+24C2m3−18C12m3+10C12m3p−16C2m3p+8C12m2p−20C12m2+16C2m2+32C2mp−16C12mp−8C12m−32C12p)en3+⋯+O(en5).

By using (9), (11), and (12), class (7) satisfies the error equation:

(13) en+1=((−32H(3)(p(m−1)/m)((−2+2p+mp)p2)3m9(2+m)2+((12−2m+2m2+2m3+m4+1536(2+m)4(−2+(2+m)p)3+192(12+m(4+m))(2+m)4(−2+(2+m)p)2+48(24+m(20+m(8+m)))(2+m)4(−2+(2+m)p))(3m5)−1))C13−C1C2m+mC3(m+2)2)en4+O(en5).

This shows that our class (7) reaches the local quartically convergence using three evaluations. The proof is complete.

The proposed class (7) uses only three functional evaluations per full cycle to achieve fourth order of convergence based on Theorem 1, which implied 4^1/3^ ≈ 1.587 as its classical computational efficiency index that is the same as (5) and (6) and is higher than 3^1/3^ ≈ 1.442 of methods (3) and (4).

A simple optimal and efficient method from class (7) could be presented in what follows:

(14) yn=xn−2mm+2f(xn)f′(xn),xn+1=yn−2f(xn)+(yn−xn)(f′(yn)+f′(xn))2f′(yn)×{H(τn)},

by choosing

(15) H(τn)=−m(2+m)p−2+2p+mp+−2m(4+m)+(2+m)3p4(−2+(2+m)p)2×(f′(yn)f′(xn)−p(m−1)/m)+m3(2+m)(−4m+4m(3+m)p−(2+m)3p2)8p(−2+(2+m)p)3×(f′(yn)f′(xn)−p(m−1)/m)2.

Another optimal and efficient method from class (7) with a simplified error equation could be presented in what follows:

(16) yn=xn−2mm+2f(xn)f′(xn),xn+1=yn−2f(xn)+(yn−xn)(f′(yn)+f′(xn))2f′(yn)×{H(τn)},

by choosing

(17) H(τn)=−m(2+m)p−2+2p+mp+−2m(4+m)+(2+m)3p4(−2+(2+m)p)2(f′(yn)f′(xn)−p(m−1)/m)+m3(2+m)(−4m+4m(3+m)p−(2+m)3p2)8p(−2+(2+m)p)3(f′(yn)f′(xn)−p(m−1)/m)2+1192p2(−2+(2+m)p)4m4×(−8m(2+m)(8+m(2+m(6+m(4+m))))+12m(2+m)(24+m(12+m(14+m(14+m(6+m)))))p−6m(192+m(192+m(136+m(124+m(94+m(42+m(10+m)))))))p2+(2+m)5(12+m(−2+m(2+m(2+m))))p3)(f′(yn)f′(xn)−p(m−1)/m)3.

This method has the simplified error equation:

(18) $$\begin{array}{c} e_{n+1}=\left(-\frac{C_{1}C_{2}}{m}+\frac{mC_{3}}{{\left(m+2\right)}^{2}}\right)e_{n}^{4}+O\left(e_{n}^{5}\right). \end{array}$$

An important challenge in the iterative methods based on the known multiplicity is to find the order of multiplicity correctly. However, most algorithms to determine the order of multiplicity may lead to mutually opposite requirements; for example,

(i) Traub in [5] showed that
  (19) $$\begin{array}{c} m\approx \frac{\log\left|f\left(x\right)\right|}{\log\left|f\left(x\right)/f^{\prime }\left(x\right)\right|}, \end{array}$$
 when *x* is very close to the multiple root of *f*;
(ii) Lagouanelle in [16] proposed the following approximate formula:
  (20) $$\begin{array}{c} m\approx \frac{f^{\prime }{\left(x\right)}^{2}}{f^{\prime }{\left(x\right)}^{2}-f\left(x\right)f^{\prime \prime }\left(x\right)}, \end{array}$$
 when once again *x* is very close to the multiple root of *f*.

The above-mentioned ways demand a very close approximation to calculate a multiplicity of high accuracy. On the other hand, to find a very close approximation to a multiple root, it is necessary to use precise multiplicity. Sometimes, both of the requirements cannot be attained at the same time.

Taking into account the opposite demands mentioned and additional calculations to find multiplicity, in those cases where we cannot provide an accurate multiplicity, it is sometimes better to apply a method which does not explicitly require the order of multiplicity, in spite of its lower computational efficiency arising from additional functional evaluations per iteration.

Hence, by applying the optimal class of methods (1) on the transformation *h*(*x*): = *f*(*x*)/*f*′(*x*), we can easily extend it for dealing with multiple roots in the absence of multiplicity, when high precision alongside high order is needed. For a simple weight function in (1) which is easily constructed by the following Mathematica command:

FullSimplify@

DSolve[{G”' [t] == 0, G[1] == 1,

G'[1] == −1/4, G”[1] == 7/4}, G[t], t];

weight[t_] = G[t]/. %[[1]]

We obtain

(21) yn=xn−23f(xn)f′(xn),xn+1=yn−2f(xn)+(yn−xn)(f′(yn)+f′(xn))2f′(yn)×{178−2tn+7tn28}, tn=f′(yn)f′(xn),

for simple zeros and the following alternative for multiple zeros in the absence of multiplicity:

(22) yn=xn+2f(xn)f′(xn)−3f′(xn)2+3f(xn)f′′(xn),xn+1=yn−((178−2ψn+7ψn28)×(f(xn)(2f′(xn)+(xn−yn)f′′(xn))f′(xn)2+(xn−yn)(−2+vn))) ×(2(1−vn))−1,

wherein

(23) $$\begin{array}{c} u_{n}=\frac{f\left(x_{n}\right)f^{\prime \prime }\left(x_{n}\right)}{f^{\prime }{\left(x_{n}\right)}^{2}},\\ v_{n}=\frac{f\left(y_{n}\right)f^{\prime \prime }\left(y_{n}\right)}{f^{\prime }{\left(y_{n}\right)}^{2}},\\ \psi_{n}=\frac{1-v_{n}}{1-u_{n}}. \end{array}$$

Therefore, now we have an efficient method (22) of order four for finding the multiple roots too. Note that until now, we have distinguished two kinds of methods: those which deal with a known order of multiplicity and others, such as (22), with no information on multiplicity.

## 4. The Dynamical Behavior of the Methods

We here investigate the comparison of iterative schemes in the complex plane using basins of attraction. It is well known that a fixed point is a point of a function that does not change under some transformation. We further recall that if we regard the evolution of a dynamical system as a series of transformations, then there may or may not be a point which remains fixed under each transformation. The final state, that a dynamical system evolves towards, corresponds to an attracting fixed point of the evolution function, but the two concepts are not equivalent because not all fixed points attract the evolution of nearby points.

The aim herein is to use the basin of attraction as another way for comparing the iteration algorithms; see, for example, [17].

In this section, we take a rectangle *D* = [−3,3]×[−3,3] ∈ *ℂ* and we assign a color to each point *z*_0_ ∈ *D* according to the multiple root at which the corresponding iterative method starting from *z*_0_ converges, and we mark the point as black if the method does not converge. In this way, we distinguish the attraction basins by their colors for different methods.

The criteria, we have used in our Mathematica codes, are that the maximum number of iterations is 100. That is to say, if the method does not reach to the considered accuracy after 100 of its full iteration steps, we allocate the black color and also consider it as Not Convergent. The considered accuracy is the obtained residual of function to be less than 10^−2^.

We have used methods (3), (5), (6), and (14) for some complex functions having multiple roots with known multiplicity.

For the first test, we have taken the following function.

**Test Problem 1**

We have

(24) $$\begin{array}{c} p_{1}\left(z\right)={\left(0.3z^{5}-Iz^{2}-0.6\right)}^{5}. \end{array}$$

**Figure 1:**
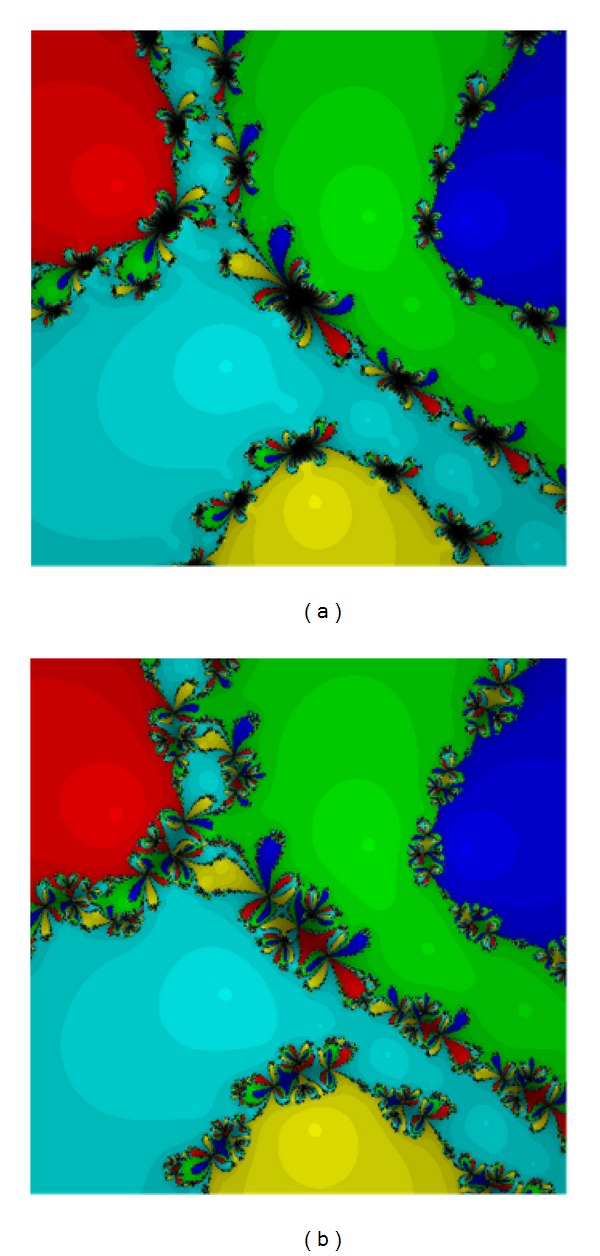
The methods (3) (a) and (5) (b) for Test Problem 1.

**Figure 2:**
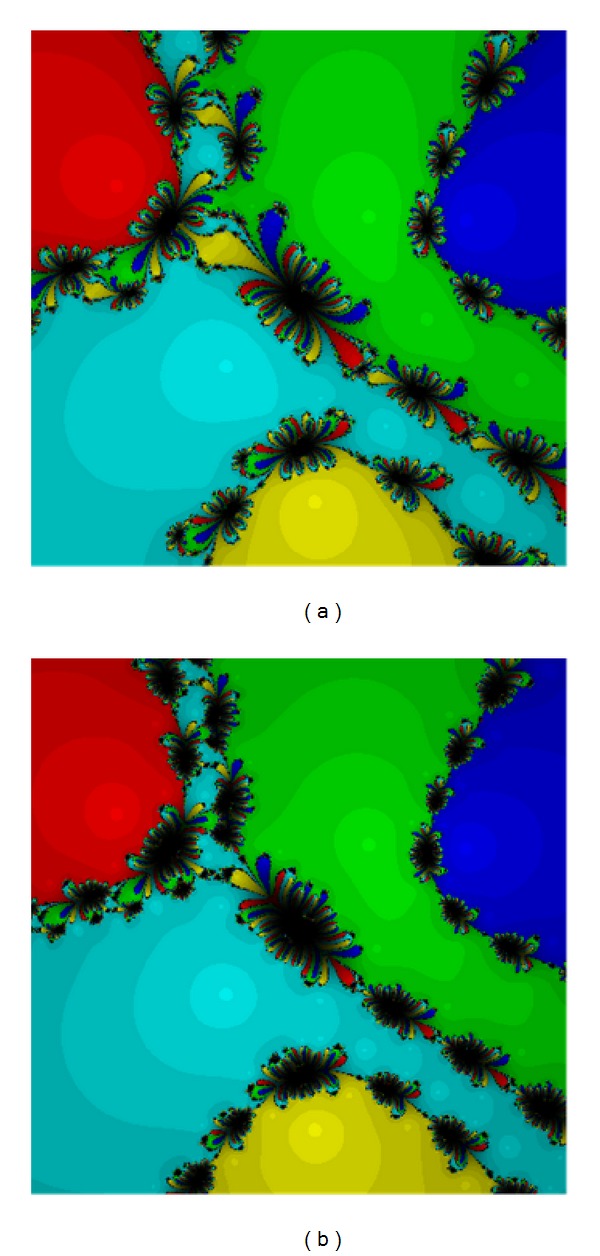
The methods (6) (a) and (14) (b) for Test Problem 1.

Equation (24) has five roots −1.35689 + 0.836942*I*, 0.121635 − 1.5236*I*, 1.24874 + 0.58223*I*, 0.527372 + 0.608843*I*, and −0.540858 − 0.504419*I* of the multiplicity five. Based on Figures 1 and 2 we can see that method (14), method (5), and method (3) are the good ones. Method (14) has larger basins in the areas corresponding to each root.

The second test problem is a polynomial as follows. Despite a few diverging points on the Julia set, we find that the basins of attraction for each root are largest for method (14).

**Test Problem 2**

We have

(25) $$\begin{array}{c} p_{2}\left(z\right)={\left(Iz^{4}-0.69z-3\right)}^{4}. \end{array}$$

**Figure 3:**
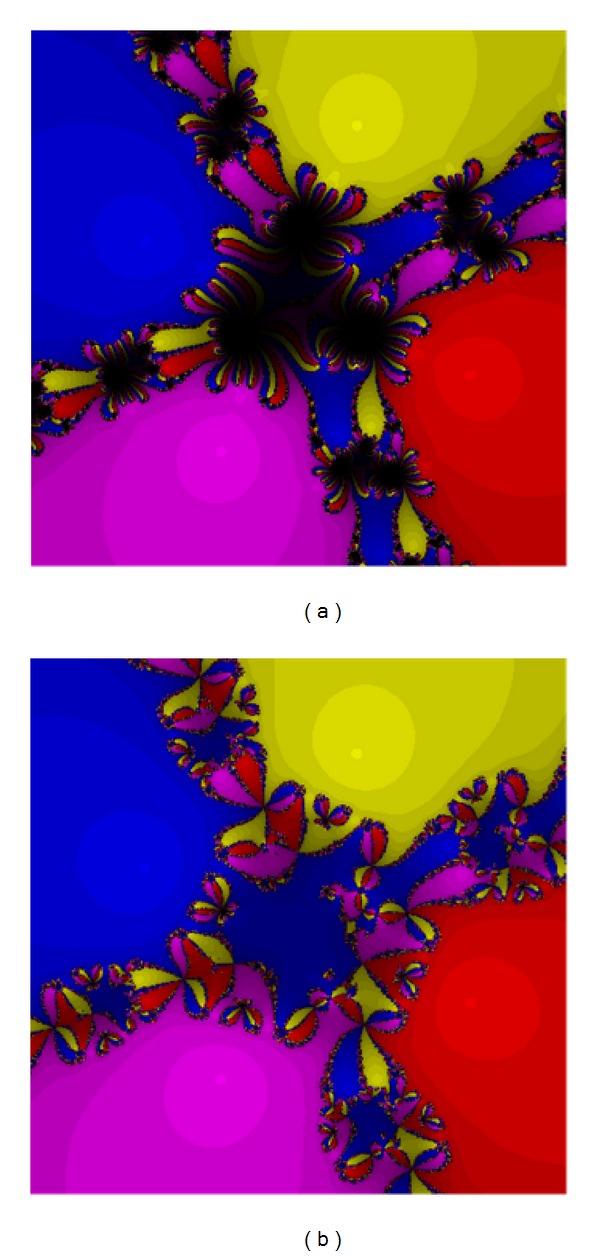
The methods (3) (a) and (5) (b) for Test Problem 2.

**Figure 4:**
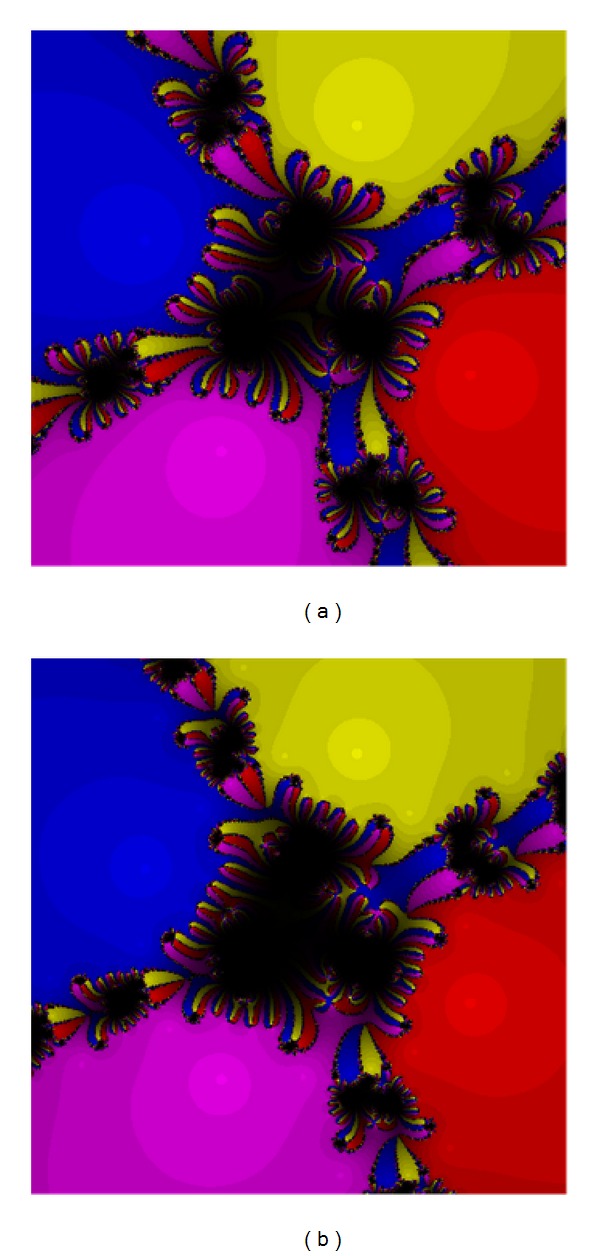
The methods (6) (a) and (14) (b) for Test Problem 2.

The zeros for this test are 1.28486 − 0.570612*I*, 0.436728@+1.28774*I*, −0.577583 − 1.1469*I*, and −1.14401 + 0.429775*I* of the multiplicity four. The results are presented in Figures 3 and 4. The performance of the methods (5) and (14) are acceptable. Methods (3), (5), (6), and (14) are quite the same though (14) has larger basins again.

The third test problem is chosen as follows.

**Test Problem 3**

We have

(26) $$\begin{array}{c} p_{3}\left(z\right)={\left(z^{6}+z\right)}^{2}. \end{array}$$

**Figure 5:**
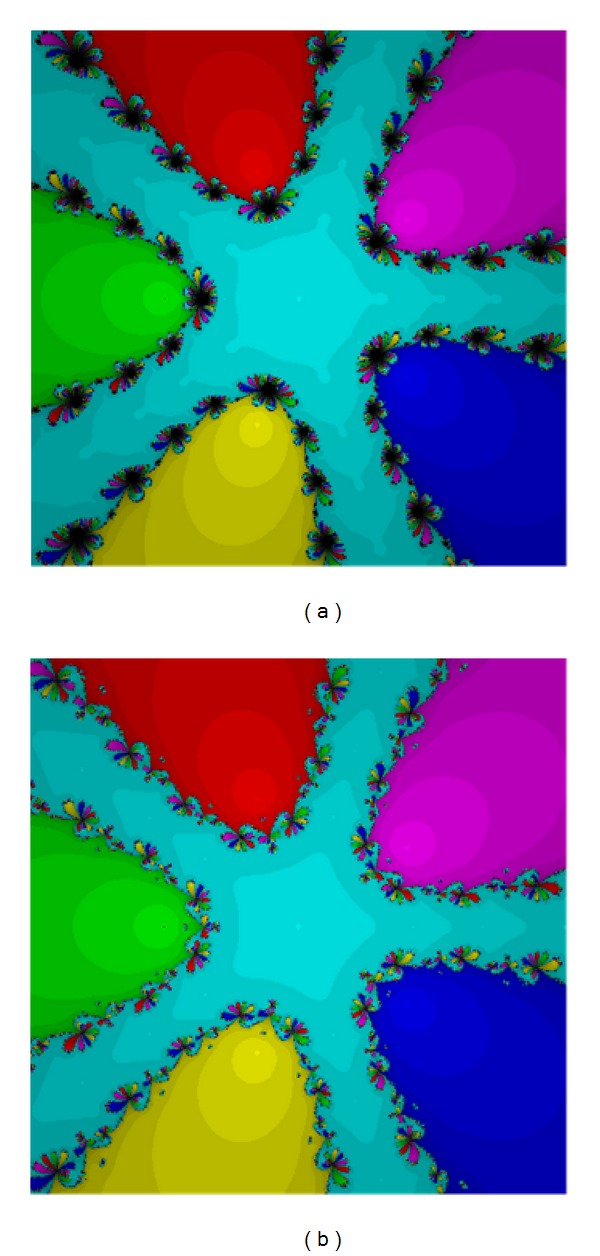
The methods (3) (a) and (5) (b) for Test Problem 3.

**Figure 6:**
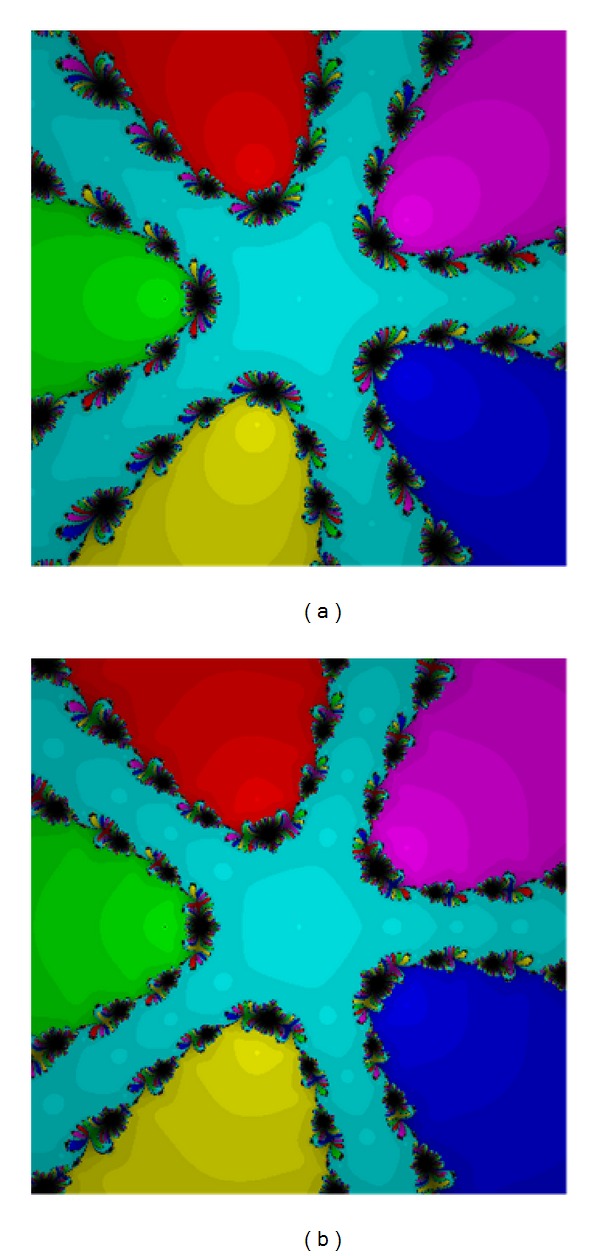
The methods (6) (a) and (14) (b) for Test Problem 3.

The roots are −0.309017 + 0.951057*I*, −0.309017 − 0.951057*I*, 0.809017 + 0.587785*I*, 0.809017 − 0.587785*I*, and −1, 0, with the known multiplicity 2. The results are presented in Figures 5 and 6. The performance of the methods (5) and (14) are acceptable. The worse methods in this case are (3) and (6).

## 5. Numerical Reports

In this section, we exhibit numerical results showing the behavior of the methods in this paper with roots of known multiplicity *m*. Iterative methods (3), (4), (5), (6), and (14) for multiple roots, which also require three functional evaluations, have been chosen. The list of test nonlinear functions including multiple zeros with their multiplicity is presented in Table 1.

The results are summarized in Tables 2 and 3 after three full iterations for two different initial guesses. As they show, the novel scheme is comparable with all of the methods. All numerical instances were performed using 500 digits floating point arithmetic. We have computed the root of each test function for the initial guess *x*_0_.

The tests show that the considered optimal methods generate results of approximately the same accuracy. The formulas in this section have been employed on several other nonlinear equations. The experience shows that there is no clear winner among the optimal methods (5), (6), and (14) in the sense that in different situations different methods may be the winners.

Following the results here and the basins of attraction and also considering the optimal efficiency index of method (14), we could conclude that class (7) is efficient and can be considered as a good tool to find multiple zeros iteratively.

Note that the application of such iterative methods for finding generalized inverses could be considered for future researches in this trend of study; see, for example, [18, 19].

## 6. Concluding Remarks

We have demonstrated the performance of a new class of fourth-order methods. Convergence analysis has proved that the new methods obtained from the class preserve their order of convergence. We do not have to evaluate the second-order derivative of the functions in the presented methods when the multiplicity is known. We have also presented a quartically convergent method in the absence of multiplicity for nonlinear equations.

Due to the fact that the basin of attraction is a method to visually comprehend how an algorithm behaves as a function of the various starting points, thus the dynamical behavior of the proposed methods alongside the comparison with the existing methods has been given in Section 4 to clearly show the efficiency of the methods.

## Figures and Tables

**Table 1:** The test functions considered in this paper.

Test functions	Zeros	Multiplicity
*f*_1_(*x*) = ((sin⁡⁡(*x*))^2^+*x*)^5^	*x*_1_* = 0	5
$f_{2}\left(x\right)={\left(1+x+\cos\left(\pi x/2\right)-\sqrt{1-x^{2}}\right)}^{3}$	*x*_2_* ≈ −0.728584046444826…	3
*f*_3_(*x*) = ((sin⁡⁡(*x*))^2^−*x* ^2^+1)^4^	*x*_3_* ≈ 1.404491648215341…	4

**Table 2:** Results of comparisons for different methods after three full iterations.

Values of functions	Guess	(3)	(4)	(5)	(6)	(14)
|*f*_1_(*x*_3_)|	0.3	0.7*e* − 80	0.8*e* − 98	0.1*e* − 164	0.3*e* − 118	0.2*e* − 164
|*f*_2_(*x*_3_)|	−0.6	0.7*e* − 72	0.1*e* − 81	0.2*e* − 151	0.4*e* − 151	0.3*e* − 151
|*f*_3_(*x*_3_)|	1.3	0.2*e* − 108	0.5*e* − 163	0.2*e* − 230	0.1*e* − 228	0.3*e* − 230

**Table 3:** Results of comparisons for different methods after three full iterations.

Values of functions	Guess	(3)	(4)	(5)	(6)	(14)
|*f*_1_(*x*_3_)|	0.2	0.2*e* − 98	0.8*e* − 120	0.7*e* − 206	0.1*e* − 128	0.8*e* − 206
|*f*_2_(*x*_3_)|	−0.8	0.3*e* − 72	0.1*e* − 111	0.7*e* − 142	0.3*e* − 141	0.9*e* − 142
|*f*_3_(*x*_3_)|	2	0.2*e* − 47	0.6*e* − 68	0.3*e* − 99	0.5*e* − 98	0.4*e* − 99
